# Clinical *Staphylococcus argenteus* Develops to Small Colony Variants to Promote Persistent Infection

**DOI:** 10.3389/fmicb.2018.01347

**Published:** 2018-06-27

**Authors:** Bei Jiang, Bo You, Li Tan, Shengpeng Yu, Han Li, Guoqing Bai, Shu Li, Xiancai Rao, Zhao Xie, Xianming Shi, Yizhi Peng, Xiaomei Hu

**Affiliations:** ^1^Institute of Burn Research, State Key Laboratory of Trauma, Burns and Combined Injury, Southwest Hospital, Third Military Medical University (Army Medical University), Chongqing, China; ^2^Department of Cardiothoracic Surgery, No. 324 Hospital of People’s Liberation Army, Chongqing, China; ^3^Department of Microbiology, College of Basic Medical Sciences, Third Military Medical University (Army Medical University), Chongqing, China; ^4^Department of Orthopedics, Southwest Hospital, Third Military Medical University (Army Medical University), Chongqing, China; ^5^Cadet Brigade, Third Military Medical University (Army Medical University), Chongqing, China; ^6^MOST–USDA Joint Research Center for Food Safety, School of Agriculture and Biology, and State Key Laboratory of Microbial Metabolism, Shanghai Jiao Tong University, Shanghai, China

**Keywords:** *Staphylococcus argenteus*, amikacin, chronic infection, persistent infection, genomic alignment

## Abstract

*Staphylococcus argenteus* is a novel staphylococcal species (also considered as a part of *Staphylococcus aureus* complex) that is infrequently reported on, and clinical *S. argenteus* infections are largely unstudied. Here, we report a persistent and recurrent hip joint infection case in which a *S. argenteus* strain and its small colony variants (SCVs) strain were successively isolated. We present features of the two *S. argenteus* strains and case details of their pathogenicity, explore factors that induce *S. argenteus* SCVs formation in the course of anti-infection therapy, and reveal potential genetic mechanisms for *S. argenteus* SCVs formation. *S. argenteus* strains were identified using phenotypic and genotypic methods. The *S. argenteus* strain XNO62 and SCV strain XNO106 were characterized using different models. *S. argenteus* SCVs were induced by the administration of amikacin and by chronic infection course based on the clinical case details. The genomes of both strains were sequenced and aligned in a pair-wise fashion using Mauve. The case details gave us important insights on the characteristics and therapeutic strategies for infections caused by *S. argenteus* and its SCVs. We found that strain XNO62 and SCV strain XNO106 are genetically-related sequential clones, the SCV strain exhibits reduced virulence but enhanced intracellular persistence compared to strain XNO62, thus promoting persistent infection. The induction experiments for *S. argenteus* SCVs demonstrated that high concentrations of amikacin greatly induce *S. argenteus* XNO62 to form SCVs, while a chronic infection of *S. argenteus* XNO62 slightly induces SCVs formation. Potential genetic mechanisms for *S. argenteus* SCVs formation were revealed and discussed based on genomic alignments. In conclusion, we report the first case of infection caused by *S. argenteus* and its SCVs strain. More attention should be paid to infections caused by *S. argenteus* and its SCVs, as they constitute a challenge to current therapeutic strategies. The problem of *S. argenteus* SCVs should be noticed, in particular when amikacin is used or in the case of a chronic *S. argenteus* infection.

## Introduction

*Staphylococcus argenteus* is a novel staphylococcal species closely related to *Staphylococcus aureus*, and is considered as a part of *S. aureus* complex (also including *Staphylococcus schweitzeri*) (Holt et al., 2011; Tong et al., 2015b; Moradigaravand et al., 2017; Olatimehin et al., 2018). It was first reported in northern Australia as a special *S. aureus* clone complex (CC) (grouped as CC75), which is highly divergent at the multiple locus sequence typing (MLST) loci compared to *S. aureus*, and therefore difficult to classify (Okuma et al., 2002; McDonald et al., 2006; Ng et al., 2009). By now, *S*. *argenteus* has been reported worldwide, but studies are still too few to reflect its clear distribution or clinical features (McDonald et al., 2006; Jenney et al., 2014; Monecke et al., 2014; Dupieux et al., 2015; Thaipadungpanit et al., 2015; Tong et al., 2015b; Argudin et al., 2016; Chantratita et al., 2016; Zhang et al., 2016; Olatimehin et al., 2018).

*S. argenteus* is phenotypically similar to *S. aureus*, and routine biochemical tests used in clinical laboratories fail to distinguish them. *S. argenteus* also has the same 16S rRNA gene sequence as *S. aureus*, thus respective sequencing will also fail (McDonald et al., 2006; Ng et al., 2009; Holt et al., 2011; Zhang et al., 2016). However, there are characteristics of *S*. *argenteus* that can be used to distinguish it from *S. aureus*, including the lack of the *crtOPQMN* operon encoding the golden pigment, a longer nonribosomal peptide synthetase (*NRPS*) gene sequence, and the different clustering in phylogenetic trees constructed based on some conserved genes (Holt et al., 2011; Zhang et al., 2016). Despite its early discovery (Okuma et al., 2002; McDonald et al., 2006; Holt et al., 2011), *S*. *argenteus* has long been neglected for some reasons. Golden pigment, an important virulence factor of *S. aureus*, is absent in all *S. argenteus* strains (Holt et al., 2011; Zhang et al., 2016). *S. argenteus* usually carries fewer exotoxin genes than *S. aureus*, and the representative *S. argenteus* strain MSHR1132 was shown to be less virulent than *S. aureus* strains in mouse models (Tong et al., 2013; Dupieux et al., 2015; Chantratita et al., 2016). In addition, *S. argenteus* strains are usually susceptible to most antimicrobials (Chantratita et al., 2016). However, several recent studies have suggested that *S. argenteus* can also result in serious infections comparable to *S. aureus* infections (Thaipadungpanit et al., 2015; Chantratita et al., 2016). Genomic analyses have revealed the pathogenic potential of *S. argenteus* and a high level of divergence in genome sequences between *S. argenteus* and *S. aureus* (Moradigaravand et al., 2017; Zhang et al., 2017). This all argues that *S. argenteus* infections should be taken more seriously, and it is necessary to distinguish *S. argenteus* and *S. aureus* infections in clinical work.

Bacterial small colony variants (SCVs) constitute a bacteria subpopulation that is characterized by small colonies (about one-tenth normal size) and changes of their metabolism (Kriegeskorte et al., 2011, 2014). Although they usually present attenuated virulence (Proctor et al., 2006; Sendi and Proctor, 2009; Kahl et al., 2016), SCVs have generated increasing interest because they are thought to be associated with recurrent and persistent infections, due to their stronger ability of intracellular persistence and decreased susceptibility to some antimicrobials compared to the parental strain (Proctor et al., 2006; Gao et al., 2010; Tuchscherr et al., 2010, 2011; Tande et al., 2014; Kahl et al., 2016). The administration of aminoglycoside antibiotics is thought to be important in SCVs formation, especially in *S. aureus*-related chronic infections (Proctor et al., 2006; Kahl et al., 2016). In laboratory experiments, SCVs result from the mutation of genes involved in electron transport or thymidine biosynthesis (Proctor et al., 2006; Melter and Radojevic, 2010). However, although several studies have reported the molecular nature of clinical SCVs, the mechanisms of SCVs formation in a clinical environment are still largely unknown (Lannergard et al., 2008; Abu-Qatouseh et al., 2010; Gao et al., 2010; Dean et al., 2014; Proctor et al., 2014; Kahl et al., 2016).

Here, we report a clinical *S. argenteus* SCV strain isolated from a patient suffering from a persistent and recurrent prosthetic hip joint infection. The parental strain (XNO62) and SCV strain (XNO106) were phenotypically compared and sequenced. Based on the case details, we analyzed and tested *in vivo* the possible reasons for the transformation from parental strain to SCVs in the course of anti-infection therapy. Finally, whole-genome comparison was used to seek possible genetic mechanisms for the formation of persistent *S. argenteus* SCV strain XNO106.

## Materials and Methods

### Ethics Statement

The study was approved by the Committee of the First Affiliated Hospital of Third Military Medical University [ethics board approval number: 2014KY(8)] and was performed in accordance with the relevant guidelines and regulations. All subjects gave written informed consent in accordance with the Declaration of Helsinki. All the personal information was removed and was not present in the data of this study.

### Bacterial Strains

*Staphylococcus argenteus* strain XNO62 and SCV strain XNO106 were obtained from a hip joint infection patient at different stages during the infection. The strains were isolated from joint fluid and periarticular tissue, respectively. Strain MSHR1132 (DSM28299), provided by Professor Xianming Shi (Shanghai Jiao Tong University, China), was used as a type strain of *S. argenteus*. *S. aureus* Newman was used as a control strain of *S. aureus*.

### Characteristics and Identification of *S. argenteus* Strains

Strains were identified as *S. argenteus* based on phenotypic and genotypic characteristics (Ghebremedhin et al., 2008; Holt et al., 2011; Tong et al., 2015b; Zhang et al., 2016). Growth curve was monitored using SpectraMax M5/M5e (Molecular Devices), overnight culture of *S. argenteus* was diluted 1:100 in TSB medium and cultured at 37°C with aeration for 24 h, OD600 values were measured every hour. The *NRPS* gene and *crtOPQMN* operon were amplified by PCR as described previously (Zhang et al., 2016). 16S rRNA and *rpoB* genes were amplified and sequenced (Ghebremedhin et al., 2008; Zhang et al., 2016). All primers used are listed in **Supplementary Table S1**. The phylogenetic tree was constructed with MEGA7 software using a neighbor-joining algorithm.

### Molecular Typing

Multiple locus sequence typing was performed as previously described in *S. aureus* (Cheng et al., 2013), except that the primers for *aroE*, *glpF*, and *yqiL* genes were designed based on the sequence of *S. argenteus* MSHR1132, as well as the primers for *spa*-typing (Tong et al., 2015b; Zhang et al., 2016). Autoinducing peptide (AIP) sequences were determined to identify the *agr* group (Monecke et al., 2010).

### Cytotoxicity Assay

Bacterial cytotoxicity was determined using a cell lifting assay as described previously (Guo et al., 2016). Briefly, 1 × 10^5^ HeLa cells were seeded into each well of a 24-well plate, cultured in DMEM medium containing 10% fetal calf serum at 37°C with 5% CO_2_ for 24 h. Exponential phase bacteria were diluted in DMEM medium and added to the cells at a multiplicity of infection (MOI) of 100. After 4 h, the medium in each well was discarded, and plates were washed twice with PBS, then stained with 200 μl 0.1% crystal violet for 10 min. The staining solution was then discarded and plates were washed twice with PBS. The cells with crystal violet dye were dissolved with 200 μl 30% acetic acid and OD490 values were measured. At least three biological replicates were performed for each group.

### Cellular Invasion and Persistence Assays

The invasion and persistence abilities of strains XNO62 and XNO106 were assessed based on previously described methods (Gao et al., 2010). Briefly, 5 × 10^4^ HeLa cells were seeded and cultured with DMEM medium containing 10% fetal calf serum in a 24-well plate, and infected by exponential phase *S. argenteus* with a MOI = 100. After 1 h incubation at 37°C with 5% CO_2_, cells were washed six times with pre-warmed PBS. Fresh medium supplemented with 400 μg/ml gentamicin was added to each well, and plates were incubated a further 48 h (gentamicin MICs for XNO62 and XNO106 are both 0.5 mg/l). The attachment/invasion was assessed before addition of gentamicin, invasion was assessed 1 h after the addition of gentamicin, and persistence was assessed at 24 and 48 h after the addition of gentamicin (Gao et al., 2010). Infected cells were lysed by 0.05% saponin to perform CFU enumeration. At least three biological replicates were performed for each group.

### Assessment of Bacterial Virulence in Mice

Female BALB/c mice aged 6–8 weeks were obtained from the Experimental Animal Center at the Third Military Medical University. All animal experiments were approved by the Institutional Animal Care and Use Committee (IACUC) of the Third Military Medical University and performed in accordance with the relevant guidelines and regulations. To assess skin abscess, 1 × 10^7^ CFU of exponential phase bacteria in 100 μl PBS was inoculated subcutaneously on the right flank. Lesion areas (A) were measured using calipers every day for a week and calculated using the formula *A* = π(*L*/2) × (*W*/2), where *L* and *W* represent the length and width of the abscess, respectively. The maximum lesion area measured during the experiment period was used for assessment of the skin infection (Malachowa et al., 2013). For the sepsis model, mice were injected with 1 × 10^8^ CFU of exponential phase bacteria in 100 μl PBS via tail vein, mice were monitored daily for 2 weeks after infection. At least 10 mice were used in each group of these experiments.

### Induction of *S. argenteus* Strain XNO62 to Form SCVs

To induce SCVs on plates, an overnight culture of the strain XNO62 was serially diluted in PBS, and plated onto TSA medium containing amikacin at 1×, 2×, 5×, 10×, and 20× MIC. After incubation at 37°C for 48 h on TSA medium, the numbers of normal and SCV colonies were counted. Three biological replicates were performed for each group.

For the *in vivo* induction experiments, mice were injected with 1 × 10^6^ CFU of exponential phase *S. argenteus* XNO62 in 100 μl PBS via the tail vein. In the amikacin induction experiments, 5 days after the infection, mice were treated with equivoluminal PBS alone or with amikacin diluted in PBS at 20, 150, or 300 mg/kg/d daily by intraperitoneal injection for another 5 days, and all mice were sacrificed at day 11 of the experiments. For the induction in chronic infection, no drug was used during the chronic infection, and mice were sacrificed until 30 days post-infection. Livers, kidneys, and femurs were homogenized and plated on TSA medium to enumerate normal and SCV colonies (Tuchscherr et al., 2016). Five mice were used in each group.

### Comparative Genomics of XNO62 and XNO106

Genomic and plasmid DNA of strains XNO62 and XNO106 were subjected to single molecule real time (SMRT) sequencing using a PacBio RS II system (performed by Novogene Co., Ltd.). The DNA sequences were aligned in a pair-wise fashion using Mauve (Darling et al., 2004).

### Statistical Analysis

Statistical analyses were performed using SPSS 19.0 (Chicago, IL, United States) and GraphPad Prism 6.0 software (San Diego, CA, United States). Continuous variables were compared using Student’s *t*-test or ANOVA. *p* < 0.05 was considered to be statistically significant.

### Accession Numbers

Strain XNO62: CP023076 for genome DNA, MH068822 for plasmid DNA. Strain XNO106: CP025023 for genome DNA, CP025250 for plasmid DNA.

## Results

### Case Details

**FIGURE 1:**
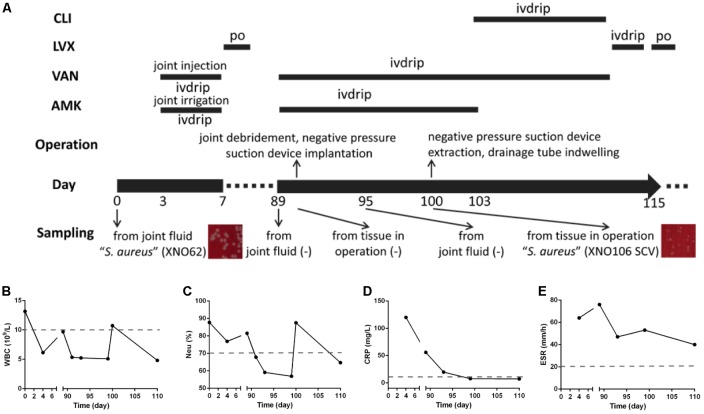
Clinical features and dynamic changes of clinical laboratory data. **(A)** The major features and events of the clinical case are presented in chronological order (day 0 and day 89 are the first and second admission dates. Information includes the timeline, administration of antimicrobials, operations, sampling for pathogenic culturing, and the results). ivdrip, intravenous drip; po, per os (oral administration). **(B**–**E)** Dynamic changes of clinical laboratory data during the course of the infection, including the values of WBC, NEU%, CRP, and ESR.

As summarized in **Figure 1**, 7 years after a revision arthroplasty of the left hip joint, a 64-year-old woman presented with increasing pain in her left hip (admitted on day 0). No sinus tract was apparent, but a 4 cm × 3 cm skin burst with crusting was observed in the anteromedial region of the left knee (unknown cause), and the surrounding skin was red and hot, with a few pustules. Bacteria isolated from the cultured aspirate (obtained from left hip joint cavity, purulent and bloody fluid) were identified as “*S. aureus*” (strain XNO62) by routine biochemical tests and 16S rRNA gene analysis. Surgical debridement was proposed, but the patient refused. Periodic dressing changes for the knee, irrigation with amikacin, injection with vancomycin for the hip joint, and systemic applications of amikacin and vancomycin were performed. The patient was discharged (day 7) after clinical symptoms improved and infection indicators declined.

On day 89, the patient was readmitted with pain and swelling in the left hip. A sinus tract and effusion of canary yellow liquid were seen in the previous operative incision. Amikacin and vancomycin were administrated intravenously and radical debridement of the hip joint was performed. Although joint fluid and tissues were sampled several times, no pathogen was found until day 100, when another “*S. aureus*” strain (strain XNO106) was isolated from the periarticular tissue in left hip during an operation. Strain XNO106 formed pinpointed colonies compared to strain XNO62, suggestive of slow growth. The patient was discharged on day 115 after clinical symptoms improved and infection indicators declined. The patient continued to have frequent relapses of pain and infection of the hip afterward. For dynamic changes of laboratory data, values of white blood cell (WBC) count, neutrophilic granulocyte percentage (Neu%), C-reactive protein (CRP), and erythrocyte sedimentation rate (ESR) were all high in the initial infection (caused by strain XNO62). In the second hospitalization, the WBC, Neu%, and CRP values were all relatively lower (increased WBC and Neu% values at day 100 might be caused by the operation), but ESR value was always high throughout the course of the infection (the patient had no hematologic disorder that may cause high ESR value).

### Strains XNO62 and XNO106 Were Identified as *S. argenteus*

**FIGURE 2:**
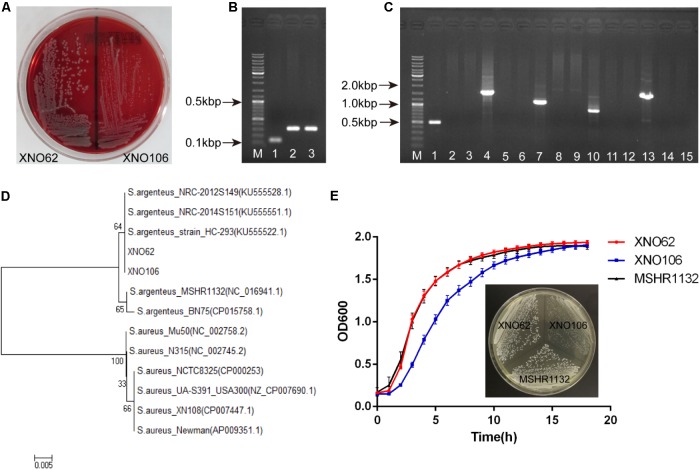
Identification of strains XNO62 and XNO106 as *S. argenteus*. **(A)** Colony morphology of strains XNO62 and XNO106 on sheep blood agar. **(B)** PCR amplicons of NRPS genes. Lane M, DNA marker; Lane 1, *S. aureus* Newman; Lane 2, strain XNO62; Lane 3, strain XNO106. **(C)** PCR amplicons of the operon *crtOPQMN*. Lane M, DNA marker; Lanes 1–3, *crtO* gene; Lanes 4–6, *crtP* gene; Lanes 7–9, *crtQ* gene; Lanes 10–12, *crtM* gene; Lanes 13–15, *crtN* gene. Lanes 1, 4, 7, 10, and 13 represent *S. aureus* Newman; Lanes 2, 5, 8, 11, and 14 represent strain XNO62; Lanes 3, 6, 9, 12, and 15 represent strain XNO106. **(D)** Neighbor-joining phylogenetic tree of strains XNO62, XNO106, and some *S. aureus* strains, based on *rpoB* gene sequences. **(E)** Growth curves and colony morphology on TSA medium for strains XNO62, XNO106, and MSHR1132.

Since we noticed that the routine molecular typing methods for *S. aureus* were not applicable for the two strains, we reasoned they may belong to *S. argenteus* strains (Ghebremedhin et al., 2008; Zhang et al., 2016). Consistent with the colony morphology of *S. argenteus* (Holt et al., 2011; Zhang et al., 2016), both strains displayed white round, non-hemolytic colonies on sheep blood agar (**Figure 2A**). A longer PCR product of the *NRPS* gene, and a lack of the *crtOPQMN* operon compared to *S. aureus* were also confirmed (**Figures 2B,C**). Although the 16S rRNA gene sequences are identical to *S. aureus* strains (data not shown), the two strains were clustered together and close to other *S. argenteus* strains in a phylogenetic tree based on *rpoB* gene sequences (**Figure 2D**). Taken together, we identified strains XNO62 and XO106 as *S. argenteus*. This is the first report of *S. argenteus* in western China.

### Strain XNO62 and SCV Strain XNO106 Are Genetically Related Sequential Clones

Strain XNO62 and SCV strain XNO106 were isolated from the same patient at different stages during the infection. Multiple molecular typing confirmed that both strains are *mecA* negative, MLST type ST2250, *spa* type t7960, and share the same AIP sequence. The gene encoding Panton–Valentine leukocidin is negative in both isolates. Mauve-based alignment demonstrated that genomic and plasmid DNA of the two strains both share more than 99% identity. These results indicate that the two strains are genetically related sequential clones, strain XNO62 is the parental strain of SCV strain XNO106.

### Characteristics of SCV Strain XNO106

Strain XNO62 had a similar growth curve as the broadly-spreading *S. argenteus* strain MSHR1132 (Holt et al., 2011), while SCV strain XNO106 exhibited a slower growth rate and pinpointed colonies (**Figure 2E**). The pinpointed colonies were observed independent of when the plated bacteria were sampled, i.e., from early or late growth stages. Strain XNO106 also retained a stable SCV phenotype for >10 passages. The amikacin MIC value for the SCV strain XNO106 increased from 4 to 8 mg/l compared to strain XNO62, while MIC values for other antimicrobials used in the patient’s anti-infection therapy did not change. In addition, strain XNO106 did not revert to normal size colonies when hemin, menadione, thymidine, tween-80, or CO_2_ was supplemented for the bacterial growth (**Supplementary Figure S1**), suggesting that new mechanisms are involved in the SCV phenotype of strain XNO106.

### SCV Strain XNO106 Exhibits Reduced Virulence and Enhanced Intracellular Persistence

**FIGURE 3:**
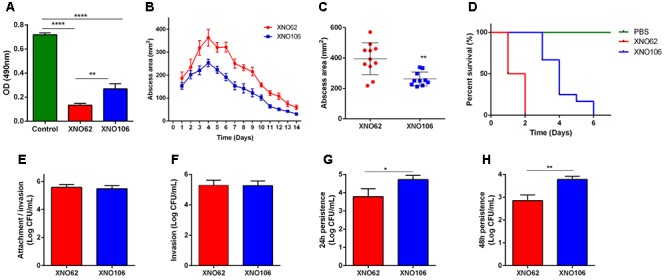
SCV strain XNO106 exhibits reduced virulence but enhanced intracellular persistence compared to strain XNO62. **(A)** Cytotoxicity of strains XNO62 and XNO106. Control, equivoluminal DMEM without bacteria. **(B,C)** Dynamic changes of abscess areas and maximal abscess areas caused by strains XNO62 and XNO106. No abscess was observed in the control group infected with PBS (not shown) (*n* ≥ 10). **(D)** Survival curves following inoculation of 1 × 10^8^ CFU bacteria via tail veins (*n* = 10). **(E**–**H)** Analyses of cellular attachment/invasion, invasion, 24 h persistence, and 48 h persistence after infection with strains XNO62 and XNO106. Bars are expressed as the mean ± SEM, by Student’s *t*-test or ANOVA. ^∗^*p* < 0.05, ^∗∗^*p* < 0.01, ^∗∗∗∗^*p* < 0.0001.

Strain XNO106 exhibited lower cytotoxicity to HeLa cells than strain XNO62 (**Figure 3A**), it also manifested smaller skin abscess areas, and reduced death rate in mice (**Figures 3B–D**). Although XNO62 and XNO106 showed similar attachment and invasion rates in HeLa cells (**Figures 3E,F**), strain XNO106 exhibited a greater persistence in cells (**Figures 3G,H**). In addition, no SCV colony was observed in the XNO62 group when colonies were observed and counted at the end of cellular assays, indicating no new SCVs were induced during the assays, and the results of these experiments properly reflect the characteristics of strains XNO62 and XNO106 themselves.

### Strain XNO62 Can Be Induced to Form SCVs by Amikacin and Chronic Infection *in Vivo*

**FIGURE 4:**
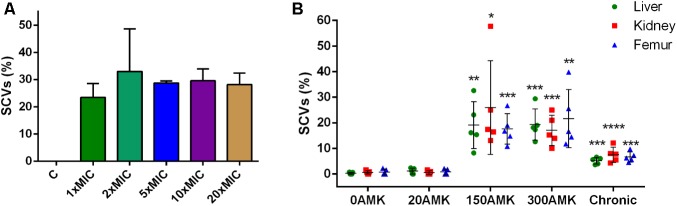
Induction of SCVs from *S. argenteus* strain XNO62. **(A)** Strain XNO62 was induced to form SCVs on TSA medium containing different concentrations of amikacin (1×, 2×, 5×, 10×, and 20× MIC). C, control group without amikacin. No significant difference for SCVs proportions among different groups was observed. **(B)** *In vivo* induction of XNO62 SCVs. Many more SCVs were recovered from the three organs of mice treated with 150 and 300 mg/kg/d AMK (150AMK and 300AMK group) compared to the control group treated with PBS (0AMK group). Chronic infections (chronic group) induced slightly more SCVs than the control group in all three organs. Bars are expressed as the mean ± SEM, *n* = 5, by Student’s *t*-test, compared to the control group (0AMK group) in each organ.^∗^*p* < 0.05, ^∗∗^*p* < 0.01, ^∗∗∗^*p* < 0.001, ^∗∗∗∗^*p* < 0.001.

Because the amikacin MIC value for SCV strain XNO106 increased from 4 to 8 mg/l compared to strain XNO62, and several studies have reported SCV formation in the course of administration of aminoglycoside antibiotics against *S. aureus* infections (Proctor et al., 2006; Melter and Radojevic, 2010), so we investigated whether amikacin could induce *S. argenteus* strain XNO62 to form SCVs. SCV formation was successfully induced when strain XNO62 was plated onto TSA containing amikacin at serial concentrations (1×, 2×, 5×, 10×, and 20× MIC). No concentration-dependent relationship was observed between amikacin concentrations and the number of SCV formation (**Figure 4A**).

Next, we tried to induce SCV formation *in vivo* by amikacin treatment and chronic infection. The SCVs percentages induced in three different organs (livers, kidneys, and femurs) were calculated in different treatment groups. The group treated with 150 or 300 mg/kg/d amikacin generated a greater number of SCVs in all the three organs, compared to the group treated with 20 mg/kg/d amikacin, or control group treated with PBS alone. The number of SCVs induced in the three organs by chronic infection was slightly, but significantly, higher than the control group, and much lower than the group treated with 150 or 300 mg/kg/d amikacin (**Figure 4B**). No significant difference in SCVs proportions was found between the groups treated with 150 and 300 mg/kg/d amikacin. The number of SCVs recovered from a single mouse liver, kidney, or femur could be very different, but there was no significant difference in proportions of SCVs among the three organs in the same treatment group (**Figure 4B**). In addition, we found that the phenotype of the induced SCVs phenotype could not be revert to normal size colonies even when hemin, menadione, thymidine, tween-80, or CO_2_ was supplemented for the bacterial growth (data not shown), indicating similar formation mechanisms in SCV strain XNO106. Taken together, these results demonstrate that chronic infection of *S. argenteus* strain XNO62 slightly induces SCV formation, while administration of high concentrations amikacin greatly induces strain XNO62 to form SCVs in different organs *in vivo*.

### Comparative Genomic Analyses Indicate Potential Mechanisms for the Reduced Growth Rate and Virulence of SCV Strain XNO106

To identify any possible genetic reason for the transition from strain XNO62 to XNO106, comparative genomic analyses were performed. 2744.503 and 2744.502 kb of genomic DNA, and 26.760 and 26.757 kb of plasmid DNA, were acquired from strains XNO62 and XNO106. No large DNA deletion or insertion was found between the two strains. Sixty-seven mutations in the genomic DNA and 24 mutations in the plasmid DNA were detected in strain XNO106 compared to XNO62. Among the genomic mutations, 47/67 mutations occurred in a prophage sequence (**Supplementary Table S2**). The 47 genomic prophage mutations and all the 24 plasmid mutations (**Supplementary Table S3**) are either intergenic mutations or mutations unrelated to bacterial growth or virulence. Among the other 20 genomic mutations, 9 were identified in noncoding regions (**Supplementary Table S4**), and most of the others are related to bacterial growth or/and virulence (**Table 1**), and therefore may be potential mechanisms for the phenotype of the SCV strain XNO106.

**Table 1:** Genomic mutations identified in ORFs of strain XNO106 relative to the parental strain XNO62.

Locus_tag^1^	Position^2^	Mutation^3^	Effect	Gene product
CJ017_01485	337359 to 337364	ATGTAT→“-”	55 Y, 56 I→“-”	PTS mannitol/fructose-specific IIA domain PtsN
CJ017_04655	946505	C→T	253 H→253 Y	Nitrite reductase [NAD(P)H] NasD
CJ017_04790	977622	“-” →GGG	“-” →P	Dithiol-disulfide isomerase DsbA
CJ017_04895	1001906 to 1001915	ACATTATTA→“-”	Frameshift	Ktr system potassium uptake protein D KtrD
CJ017_06190	1272860	C→“-”	Frameshift	Mannosyl-D-glycerate transport repressor MngR
CJ017_06625	1358853 to 1358855	AAT→“-”	Frameshift	DNA topoisomerase IV, subunit A
CJ017_06945	1428248	“-” →C	Frameshift	C-terminal processing protease CtpA
CJ017_08605	1808212 to 1808222	ATGCCAATAAA→“-”	Frameshift	Uncharacterized MFS-type transporter
CJ017_12195	2473358	“-” →C	33 D→STOP	Hypothetical protein
CJ017_12300	2496136 to 2496147	TGCCCGGCTCAC→“-”	943 S, 944 E, 945P, 946G→“-”	Fibronectin-binding protein A
CJ017_12610	2556284	“-” →TTC	“-” →R	Hypothetical protein

^1^*Locus tag of strain XNO62 genome. ^*2*^Position on strain XNO62 genome; ^*3*^“-”, deleted position*.

## Discussion

Since first discovered in 2006 (McDonald et al., 2006), *S. argenteus* infections have been increasingly reported worldwide (McDonald et al., 2006; Jenney et al., 2014; Monecke et al., 2014; Thaipadungpanit et al., 2015). In China, *S. argenteus* was first and only reported in 2016 in two cities of Eastern China (Zhang et al., 2016). In that study, 6/839 (0.72%) of the presumptive *S. aureus* isolates were identified as *S. argenteus*. Here, we presented the first report of an *S. argenteus* infection in Western China. More detailed study focused on prevalence of *S. argenteus* in China is needed in the future. It has long been believed that *S. argenteus* is a kind of hypovirulent bacteria (McDonald et al., 2006; Holt et al., 2011). But as presented in this study, we found that the two *S. argenteus* strains caused serious invasive injury in the joint, despite the administration of antibiotics presumed to be effective against *S. argenteus*. Furthermore, it is worth noting that two recent studies have shown that ST 2250 *S. argenteus* (identical to the MLST type of strains XNO62 and XNO106) can be highly prevalent, and may be associated with invasive infections (Thaipadungpanit et al., 2015; Chantratita et al., 2016). These results should sound the alarm on *S. argenteus*-related infections, especially for ST 2250 *S. argenteus*. In addition, the genome-based analyses in two recent studies also support this opinion (Moradigaravand et al., 2017; Zhang et al., 2017).

To date, only few clinical *S. argenteus* infection cases have been described. Our study provides a lesson on *S. argenteus*-related infections and therapeutic strategies. The skin infection in the left knee presented typical characteristics consistent with *S. aureus* skin infections that sometimes can spread to distant sites via blood (Tong et al., 2015a). Although bacteria isolation was not performed from the knee wound, it was very likely that the skin infection in the knee was the source of the infection, and *S. argenteus* may exhibit similar features as *S. aureus* in skin infections. The dynamic changes in clinical laboratory data over the course of infection illustrate some distinguishing features. During the first hospitalization, the patient presented with an infection of strain XNO62, distinguished by high levels of WBC, Neu%, CRP, and ESR values. Upon being admitted a second time, XNO62 was not detected but XNO106 was, and the WBC, Neu%, and CRP values all declined, while the ESR values were always high throughout the course of the infection. These data may provide possible indicators for *S. argenteus* SCVs infections. During the first hospitalization, an operative treatment was proposed, but the patient refused. This may be an important reason resulting in the chronic and persistent infection for the patient. In order to achieve a better prognosis, radical surgical debridement combined with early and sufficient administration of antimicrobials should be highly recommended for this kind of patients. Amikacin is a highly favored aminoglycoside antibiotic because it is a powerful bactericidal drug (Maurin and Raoult, 1994). However, many studies have reported an association between exposure to aminoglycosides and SCV formation, especially in cases of chronic infections (Sendi and Proctor, 2009; Melter and Radojevic, 2010). In our case, amikacin was frequently used as the anti-infection therapy, and we found that SCV strain XNO106 had higher MIC values for amikacin than the parental strain XNO62. Of note, SCV strain XNO106 was isolated at an early stage of SCV occurrence throughout the course of the infection, which may be why the MIC value is not still higher. Nevertheless, it was already capable of promoting a persistent infection and possibly resulting in a more serious threat if the infection was allowed to continue. We verified that amikacin can induce strain XNO62 to form *S. argenteus* SCVs *in vitro* and *in vivo*. The increased MIC value makes strain XNO106 less susceptible to amikacin, and the increased intracellular persistence makes it difficult to detect, and facilitates its evasion of both antibiotic killing and immune clearance. These results suggest that amikacin should be used with care in *S. argenteus* infections, particularly for those that tend to develop chronic and refractory infections. When infection symptoms persist in spite of administration of susceptible antimicrobials, and no pathogen is detected even after multiple sampling, a SCV infection should be taken into consideration.

In our *in vitro* study, different concentrations of amikacin induced similar percentages of *S. argenteus* SCV formation on plates. While *in vivo*, high doses of amikacin induced much more *S. argenteus* SCV formation than the low dose or chronic infection. This indicates different SCV induction modes in different environmental factors. SCVs are related to many clinical diseases involving in different body sites, mainly including osteomyelitis, cystic fibrosis, and device-related infections (Proctor et al., 2006; Melter and Radojevic, 2010). In this study, we compared the SCVs percentages recovered from different organs under different induction conditions, the results suggest that *S. argenteus* SCV formation could occur in all the tested organs including liver, kidney, and femur. Administration of high concentrations amikacin greatly induces *S. argenteus* SCVs formation, chronic infection also induces the SCV formation, although at a relatively lower level. So the issue of *S. argenteus* SCVs should be stressed in infections of various body sites, especially in the cases of amikacin administration and chronic infection.

The molecular nature of clinical SCVs has been reported in several studies, such as mutations of the *menB* gene and the differential expression of non-protein-coding RNAs (Lannergard et al., 2008; Abu-Qatouseh et al., 2010). However, the genetic mechanisms for clinical SCVs formation are still largely unknown, and *S. argenteus* SCV strain is first reported in this study (Gao et al., 2010; Melter and Radojevic, 2010; Dean et al., 2014; Proctor et al., 2014; Kahl et al., 2016). We found some genetic variations that might be responsible for the phenotype of *S. argenteus* SCV strain XNO106 (**Table 1**). The bacterial phosphotransferase system (PTS) couples the transport of carbohydrates with their simultaneous phosphorylation (Lengeler et al., 1990). A *ptsN* gene deletion (encoding the PTS mannitol/fructose-specific IIA domain) results in SCV formation in *Sinorhizobium meliloti* and *Brucella melitensis* (Pinedo et al., 2008; Dozot et al., 2010). A link between enzyme IIA and bacterial virulence has also been observed in several bacterial species (Kok et al., 2003; Zeng and Burne, 2010). The *ptsN* mutation in strain XNO106 (deletion of 55 Y and 56 I) occurs in the phosphorylation active site of the protein, further suggesting its important role in the XNO106 SCV phenotype. MngR is a repressor of *mngA*, which encodes a mannosyl-D-glycerate-specific PTS EIIABC component (Sampaio et al., 2004), so the mutation in *mngR* may also affect bacterial growth and virulence. NasD catalyzes the generation of NAD(P)H from NAD(P)^+^ (Shi et al., 2014). Since the TCA cycle is important for bacterial energy metabolism and it is also the primary consumer of NAD(P)^+^, a mutation in *nasD* may affect bacterial energy metabolism by altering the NAD(P)^+^ concentration, and thus affecting bacterial growth (Kriegeskorte et al., 2011, 2014). DsbA is the primary catalyst of proteins’ disulfide bond formation, and had been associated with pathogenicity in Gram-negative bacteria (Heras et al., 2009). But reduced virulence was not observed when *dsbA* was deleted in *S. aureus* (Dumoulin et al., 2005), so its role in *S. argenteus* remains to be elucidated. Intracellular K^+^ is important for bacterial virulence (Su et al., 2009). Ktr is a specific potassium uptake system in *S. aureus* (Grundling, 2013), so the mutation in *ktrD* may contribute to reduced bacterial virulence. DNA topoisomerase IV can decatenate DNA following DNA replication, thus facilitating segregation of DNA into daughter cells (Rawdon et al., 2016). Deficiency of topoisomerase IV may lead to SCV formation due to dysfunction of cell division. CtpA is a C-terminal processing serine protease. A *ctpA* deletion strain of *S. aureus* is attenuated for virulence in mice (Carroll et al., 2014), the *ctpA* mutation in XNO106 may have a similar effect. However, additional experiments are required to confirm the effects of these mutations, the results may help identify new strategies to defeat pathogenic *S. argenteus* SCVs.

In summary, we studied a prosthesis-related infection caused by a *S. argenteus* strain and its SCV strain. The case details shed light on the consequences of some anti-*S. argenteus* therapeutic strategies, and the characteristics of infection caused by *S. argenteus* and its SCVs. Amikacin and chronic infections were proven to induce *S. argenteus* SCV formation, thus promoting persistent infection. Potential mechanisms for *S. argenteus* SCV formation were discussed. We recommend that the problem of *S. argenteus* SCVs be recognized when amikacin is used for anti-*S. argenteus* therapy or in a case of chronic *S. argenteus* infection, and this kind of infections may form a challenge to the current therapeutic strategies. Clinical infections caused by *S. argenteus* and its SCVs should get more attention.

## Author Contributions

XH and YP conceived and designed this study. BJ, BY, LT, SY, HL, and GB carried out the experiments and analyzed the results. SL, XR, and ZX helped with the data interpretation. XS provided strain MSHR1132 and gave important suggestions on design of the study. BJ, XH, and YP drafted the manuscript. All authors have read and approved the final manuscript.

## Conflict of Interest Statement

The authors declare that the research was conducted in the absence of any commercial or financial relationships that could be construed as a potential conflict of interest.

## Supplementary Material

FIGURE S1 Auxotrophy studies for *S. argenteus* XNO106. Strain XNO62 and strain XNO106 were streaked on TSA, TSA + hemin (10 μg/ml), TSA + menadione (25 μg/ml), TSA + thymidine (125 μg/ml), or TSA + tween80 (0.1%), and incubated aerobically at 37°C for 24 h. Both strains were also streaked on TSA and incubated at 37°C for 24 h in an atmosphere containing 5% CO_2_. XNO106 colonies failed to revert to normal size with all the treatment factors above.

TABLE S1 Primers used in this study.

TABLE S2 Mutations identified in the prophage regions of XNO106 genome relative to XNO62.

TABLE S3 Mutations identified in the plasmid of strain XNO106 relative to the plasmid of parental strain XNO62.

TABLE S4 Mutations identified in the noncoding regions of XNO106 genome relative to XNO62.
