# Supplementary figures and images for: Clinical Staphylococcus argenteus Develops to Small Colony Variants to Promote Persistent Infection
Source: Front Microbiol. 2018 Jun 27;9:1347. doi: 10.3389/fmicb.2018.01347 (PMC6036243; doi:10.3389/fmicb.2018.01347)

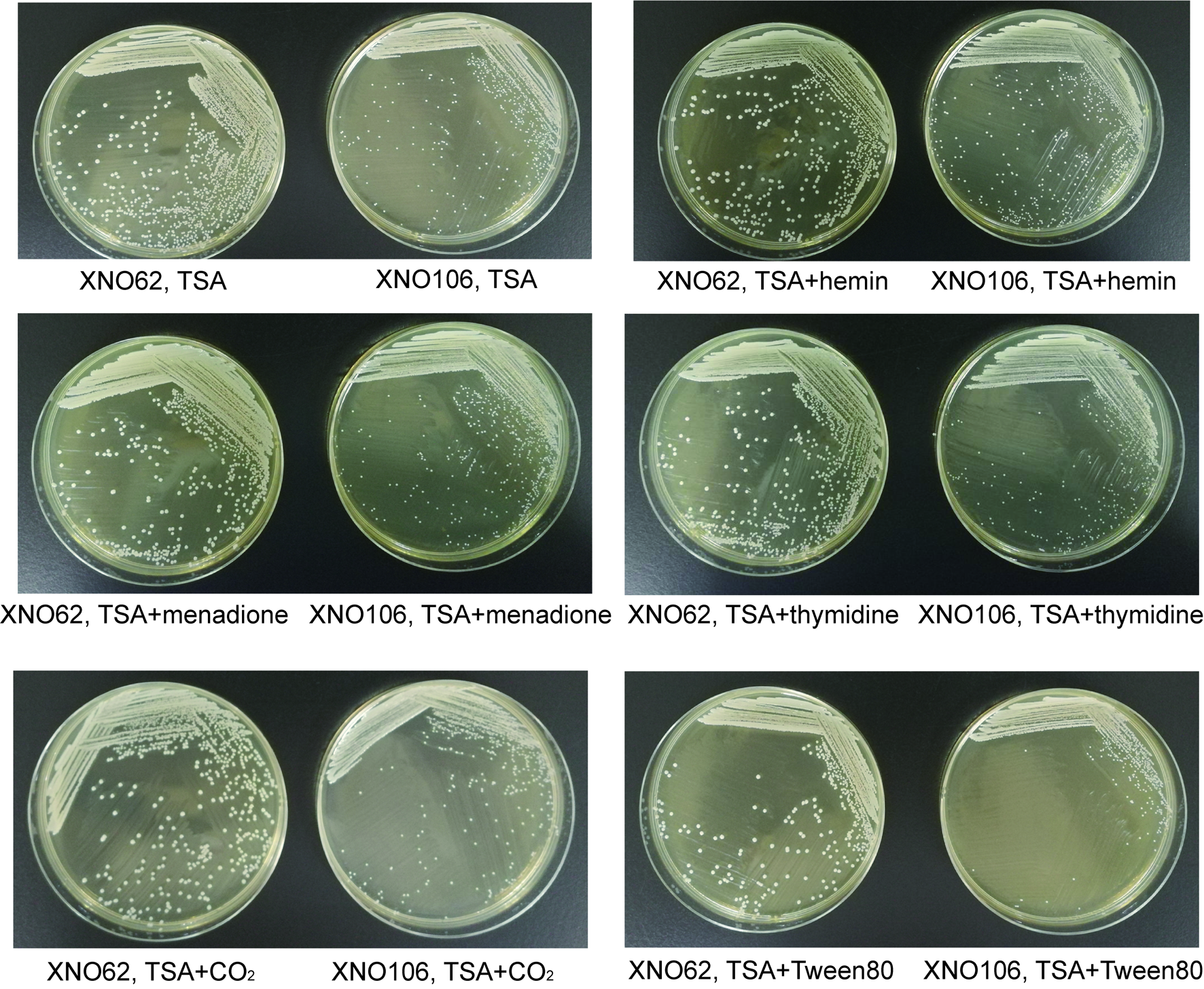

Supplement: FIGURE S1 — Auxotrophy studies for S. argenteus XNO106. Strain XNO62 and strain XNO106 were streaked on TSA, TSA + hemin (10 μg/ml), TSA + menadione (25 μg/ml), TSA + thymidine (125 μg/ml), or TSA + tween80 (0.1%), and incubated aerobically at 37°C for 24 h. Both strains were also streaked on TSA and incubated at 37°C for 24 h in an atmosphere containing 5% CO2. XNO106 colonies failed to revert to normal size with all the treatment factors above. [file Image_1.TIF]
