# Supplementary material for: Clinical Staphylococcus argenteus Develops to Small Colony Variants to Promote Persistent Infection
Source: Front Microbiol. 2018 Jun 27;9:1347. doi: 10.3389/fmicb.2018.01347 (PMC6036243; doi:10.3389/fmicb.2018.01347)
Supplement: TABLE S1 — Primers used in this study. [file Table_1.PDF]

Table S1. Primers used in this study

Primers	Sequences(5’-3’)
crtO-F	ATGAAAACCATGAAAAAATATATT
crtO-R	TTAGTCATGACGTTCACCTTTTC
crtP-F	ATGACTAAACATATCATCGTTATTGG
crtP-R	TCACTTCCTATTCTTCGCTTC
crtQ-F	ATGAAATGGTTATCACGAAT
crtQ-R	TTATTGTTCTTTAGATATAGAATATTG
crtM-F	ATGACAATGATGGATATGAAT
crtM-R	CTATATTCTATGATATTTACTATTTATTT
crtN-F	ATGAAGATTGCAGTAATTGGTGC
crtN-R	TTATACGCCCCGCTCAATAT
16s 27F	AGAGTTTGATCMTGGCTCAG
16s 907R	CCGTCAATTCMTTTRAGTTT
rpoB-2491F	AACCAATTCCGTATIGGTTT
rpoB-3241R	GCIACITGITCCATACCTGT
nrps-F	TTGARWCGACATTACCAGT
nrps-R	ATWRCRTACATYTCRTTATC
