# Supplementary material for: Clinical Staphylococcus argenteus Develops to Small Colony Variants to Promote Persistent Infection
Source: Front Microbiol. 2018 Jun 27;9:1347. doi: 10.3389/fmicb.2018.01347 (PMC6036243; doi:10.3389/fmicb.2018.01347)
Supplement: TABLE S2 — Mutations identified in the prophage regions of XNO106 genome relative to XNO62. [file Table_2.PDF]

Table S2. Mutations identified in the prophage regions of XNO106 genome relative to XNO62.

Gene locus	Position[1]	Mutation[2]	Effect	Gene product
CJ017_04170	858063	"-" →T	230E→STOP	conserved phage family protein / hypothetical protein
CJ017_04175	858197	"-" →A	Frameshift	putative phage DnaC-like protein (DNA replication protein DnaC [Replication, recombination and repair])
CJ017_04190	859477	"-" →A	Frameshift	phage protein (Protein of unknown function (DUF1064),This family consists of several phage and bacterial proteins of unknown function)
CJ017_04200	859978	"-" →A	Frameshift	PVL phage protein
CJ017_04200	860199	T→"-"	Frameshift	PVL phage protein
CJ017_04215	861086	T→"-"	Frameshift	hypothetical protein
CJ017_04240	862691	"-" →A	Frameshift	phage transcriptional activator, RinA family
CJ017_04240	862762	"-" →T	Frameshift	phage transcriptional activator, RinA family
CJ017_04240	862790	A→"-"	Frameshift	phage transcriptional activator, RinA family
CJ017_04240	862830	"-" →T	Frameshift	phage transcriptional activator, RinA family
CJ017_04240	862860	A→"-"	Frameshift	phage transcriptional activator, RinA family
CJ017_04240	862897	G→"-"	Frameshift	phage transcriptional activator, RinA family
-	862948	A→"-"	Intergenic	-
-	862998	G→"-"	Intergenic	-
-	863004	"-" →A	Intergenic	
CJ017_04245	863293	C→"-"	Frameshift	bacteriophage terminase small subunit
CJ017_04245	863413	"-" →A	Frameshift	bacteriophage terminase small subunit

CJ017_04250	864033	A→“-”	Frameshift	bacteriophage terminase large subunit
CJ017_04255	864893	“-” →A	Frameshift	phage portal protein, SPP1 family
CJ017_04255	864912	“-” →A	Frameshift	phage portal protein, SPP1 family
CJ017_04255	864960	“-” →A	Frameshift	phage portal protein, SPP1 family
CJ017_04255	866265	“-” →A	Frameshift	phage portal protein, SPP1 family
CJ017_04260	866800	“-” →A	Frameshift	putative phage head morphogenesis protein
CJ017_04265	867594	“-” →A	Frameshift	hypothetical protein
-	867652	“-” →T	Intergenic	-
-	867720	“-” →T	Intergenic	-
CJ017_04270	868401	“-” →A	Frameshift	Phage_capsid domain-containing protein
CJ017_04270	868653	“-” →A	Frameshift	Phage_capsid domain-containing protein
CJ017_04270	868739	“-” →T	Frameshift	Phage_capsid domain-containing protein
CJ017_04280	869496	“-” →A	Frameshift	phage terminase small subunit
CJ017_04280	869573	“-” →A	Frameshift	phage terminase small subunit
CJ017_04280	869648	“-” →A	Frameshift	phage terminase small subunit
CJ017_04285	869700	“-” →A	Frameshift	Phage gp6-like head-tail connector protein
CJ017_04285	869735	“-” →A	Frameshift	Phage gp6-like head-tail connector protein
CJ017_04285	869812	“-” →C	Frameshift	Phage gp6-like head-tail connector protein
CJ017_04290	870176	“-” →T	Frameshift	hypothetical protein
CJ017_04290	870209	“-” →T	Frameshift	hypothetical protein
CJ017_04295	870438	“-” →C	Frameshift	phage protein, HK97

				gp10 family
CJ017_04295	870635	“-” →G	Frameshift	phage protein, HK97 gp10 family
CJ017_04300	870924	“-” →A	Frameshift	hypothetical protein
CJ017_04300	870942-870943	CA→AC	91T→91N	hypothetical protein
CJ017_04300	871052	“-” →A	Synonymous	hypothetical protein
CJ017_04305	871152	A→“-”	29L →STOP	Phage_tail_2 domain-containing protein
CJ017_04305	871188	“-” →T	Frameshift	Phage_tail_2 domain-containing protein
CJ017_04305	871233	“-” →T	Frameshift	Phage_tail_2 domain-containing protein
CJ017_04305	871245	“-” →A	Frameshift	Phage_tail_2 domain-containing protein
CJ017_04305	871270	“-” →T	Frameshift	Phage_tail_2 domain-containing protein

[1]position on strain XNO62 genome; [2]-, deletion.
