# Supplementary material for: Clinical Staphylococcus argenteus Develops to Small Colony Variants to Promote Persistent Infection
Source: Front Microbiol. 2018 Jun 27;9:1347. doi: 10.3389/fmicb.2018.01347 (PMC6036243; doi:10.3389/fmicb.2018.01347)
Supplement: TABLE S3 — Mutations identified in the plasmid of strain XNO106 relative to the plasmid of parental strain XNO62. [file Table_3.pdf]

Table S3. Mutations identified in the plasmid of strain XNO106 relative to the plasmid of parental strain XNO62

Gene locus1	Position[2]	Mutation[3]	Effect	Gene product
CJ017_p0130	26733	"-" →A	Frameshift	Adenosylmethionine decarboxylase
-	26413	A→"-"	Intergenic	-
-	26298	A→T	Intergenic	-
-	26296	T→A	Intergenic	-
-	26291	T→"-"	Intergenic	-
-	26173	"-" →A	Intergenic	-
-	26120	"-" →A	Intergenic	-
XNO62.plasGM000002	26062	T→"-"	STOP→E	Regulatory protein BlaR1
-	25747	T→"-"	Intergenic	-
-	25595	"-" →G	Intergenic	-
-	25557	T→"-"	Intergenic	-
CJ017_p0120	24975	T→"-"	Frameshift	Beta-lactamase
-	24869	T→"-"	Intergenic	
CJ017_p0115	24664	G→"-"	16P→16G, 17I→STOP	Putative beta-lactamase
-	24368	"-" →C	Intergenic	-
CJ017_p0105	23765	"-" →C	Frameshift	Conserved hypothetical protein 698
-	21743	A→"-"	Intergenic	-
-	21424	T→G	Intergenic	-
-	21423	G→C	Intergenic	-
-	21421	C→A	Intergenic	-
-	21367	"-" →T	Intergenic	-
-	21196	"-" →T	Intergenic	-
-	20388	G→"-"	Intergenic	-
CJ017_p0080	17767	C→"-"	Frameshift	Cadmium resistance family protein

[1]locus tag of XNO62 plasmid; [2] position on strain XNO62 plasmid [3]-, deletion.
