# Supplementary material for: Clinical Staphylococcus argenteus Develops to Small Colony Variants to Promote Persistent Infection
Source: Front Microbiol. 2018 Jun 27;9:1347. doi: 10.3389/fmicb.2018.01347 (PMC6036243; doi:10.3389/fmicb.2018.01347)
Supplement: TABLE S4 — Mutations identified in the noncoding regions of XNO106 genome relative to XNO62. [file Table_4.PDF]

Table S4. Mutations identified in the noncoding regions of XNO106 genome relative to XNO62.

Position[1]	Mutation[2]	Effect
299916	"-" →G	Intergenic
465314	"-" →GGT	Intergenic
723864	"-" →G	Intergenic
1705940	"-" →C	Intergenic
2091522	T→"-"	Intergenic
2191608	"-" →G	Intergenic
2091633	"-" →C	Intergenic
2091666	"-" →T	Intergenic
2091945-2091946	CA→AC	Intergenic

[1], position in strain XNO62 genome; [2]-, deletion.
